# Neurodevelopmental impact of early diagnostic imaging in preterm infants: quantifying risk and the role of point-of-care ultrasound

**DOI:** 10.3389/fped.2025.1642629

**Published:** 2025-08-20

**Authors:** Indrani Bhattacharjee, MaryAnn Volpe, Siddharth Bhattacharya, Cecelia Sibley, Paige Church

**Affiliations:** ^1^ Division of Newborn Medicine, Tufts Medical Center, Boston, MA, United States; ^2^ Department of Pediatrics, Tufts University School of Medicine, Boston, MA, United States; ^3^ Department of Management Information Systems, George Mason University, Fairfax, VA, United States; ^4^ Department of Pediatrics, Boston Children’s Hospital, Boston, MA, United States

**Keywords:** preterm infants, neurodevelopment, NICu, x-ray exposure, Bayley-III, SNAPPE-II, neonatal imaging, cumulative radiation

## Abstract

**Background:**

Medical imaging is essential in neonatal intensive care units (NICUs), particularly for the management of preterm infants. However, concerns persist regarding the neurodevelopmental impact of repeated low-dose radiation exposure. This study aimed to investigate whether cumulative x-ray exposure in the first month of life is associated with neurodevelopmental outcomes in preterm infants.

**Methods:**

We conducted a retrospective chart review of preterm infants (<34 weeks gestation) admitted to the Level IIIB NICU at Tufts Medical Center. Infants were included if they had at least one x-ray within the first 24 h of life and were followed consistently at the neurodevelopmental clinic till 12–18 months corrected age. Exclusion criteria included major congenital anomalies, severe perinatal complications and loss to follow-up. Cumulative x-ray exposure was recorded at Day 1, Day 7, and Month 1. Neurodevelopment was assessed using Bayley Scales of Infant and Toddler Development, Third Edition (Bayley-III). Multiple linear regression analyses were used to assess associations, adjusting for gestational age, birth weight, comorbidities and SNAPPE-II scores.

**Results:**

Among 53 infants, cumulative imaging by Day 7 and Month 1 was significantly associated with lower Bayley-III motor and cognitive scores. Each additional x-ray by Day 7 was associated with a 1.38-point decline in motor scores (*p* < 0.001) and a 0.89-point decline in cognitive scores (*p* = 0.046). These associations persisted at Month 1. No significant effects were found for imaging on Day 1. Language outcomes showed non-significant downward trends.

**Conclusion:**

Frequent x-ray exposure in the first month of life may be associated with worse motor and cognitive development in preterm infants. These findings suggest the need for weight-based diagnostic reference levels in NICUs and support incorporation of alternative imaging such as point of care ultrasound (POCUS into routine neonatal intensive care.

## 1 Introduction

Advancements in neonatal intensive care have dramatically improved survival among preterm infants, but this progress has come with new challenges. Among the most pressing is the need to balance life-saving diagnostic imaging with the risks of cumulative radiation exposure. Neonates, particularly those born extremely preterm, are subjected to frequent chest and abdominal x-rays during their NICU stay, often within the first days or weeks of life. These imaging studies, though clinically essential, expose fragile, rapidly developing tissues—especially the brain—to ionizing radiation at a time when neurogenesis, synaptogenesis, and myelination are at their peak.

Numerous studies have raised concerns about the safety of repeated imaging in neonates. For example, Crealey et al. (1) reported that very low birth weight (VLBW) and extremely low birth weight (ELBW) infants undergo multiple radiographs daily, a pattern driven by their clinical instability and prolonged hospitalization. Weiß et al. (2) quantified cumulative thoracic and abdominal radiographic doses and found that exposure levels often exceeded international safety recommendations, especially in high-risk infants. Despite such data, the long-term neurodevelopmental impact of this early-life radiation remains poorly understood.

Radiobiological evidence suggests that the developing brain is particularly vulnerable to ionizing radiation, which can induce oxidative stress, white matter injury, and apoptosis of neural progenitor cells, potentially disrupting motor and cognitive development (3, 4). However, clinical studies have historically focused more on cancer risk (5, 6) than on neurodevelopmental consequences, leaving a critical gap in our understanding. In this context, the present study investigates the relationship between cumulative neonatal x-ray exposure and neurodevelopmental outcomes, as measured by Bayley-III scores at 12–18 months of age.

## 2 Methodology

This retrospective chart review was conducted in the Level IIIB Neonatal Intensive Care Unit (NICU) at Tufts Medical Center and was approved by the Tufts Health Sciences Institutional Review Board. The chart review included infants admitted between January 2018 and December 2020. The study population included preterm infants born at less than 34 weeks of gestation who were admitted to the NICU for a duration exceeding 48 h and who received at least one x-ray within the first 24 h of life. Infants were excluded if they had major congenital anomalies, genetic syndromes, severe perinatal asphyxia, high-grade intraventricular hemorrhage (Grade III or IV), cystic periventricular leukomalacia, hemodynamically significant patent ductus arteriosus (HsPDA) needing device closure or more than one pharmacologic approach, culture-positive sepsis or meningitis and necrotizing enterocolitis (NEC) classified as Stage IIb or higher. In addition, infants who missed two consecutive neurodevelopmental follow-up visits within six months of NICU discharge were excluded from the analysis. These exclusion criteria were designed to minimize the influence of known confounders that could independently influence neurodevelopmental outcomes.

Demographic and clinical data were extracted from the infants' electronic medical records. Key variables collected included gestational age, birth weight, and the Score for Neonatal Acute Physiology with Perinatal Extension-II (SNAPPE-II), which was used to quantify illness severity within the first 12 h of life. Cumulative x-ray exposure was recorded at three specified time points: Day 1, Day 7, and Day 30 (Month 1) of life. Radiographs in our NICU are ordered based on clinical judgment and indication. Common indications include confirmation of line or tube placement, respiratory status assessment, abdominal distension, and evaluation for suspected sepsis or NEC. There is no fixed protocol mandating routine imaging at specific intervals; instead, imaging frequency varies based on clinical acuity, aligning with common practice in most NICUs (17, 20, 22).

Direct measurements of radiation dose were not available in this retrospective chart review. Instead, cumulative x-ray exposure was estimated by the total number of radiographic studies each infant received. This included both chest and abdominal radiographs and was used as a surrogate for radiation burden. While this approach lacks granularity regarding dose intensity, it reflects real-world imaging frequency and clinical burden.

Due to the retrospective design and the frequent anatomical overlap in radiographic fields—particularly in preterm neonates with limited thoracoabdominal surface area—we did not distinguish between chest and abdominal radiographs in our quantification. All imaging events were included in the cumulative count irrespective of anatomical intent. This reflects a common scenario in neonatal imaging, where despite efforts to optimize collimation and target specific organ systems, inadvertent exposure of adjacent structures is often unavoidable due to the small body habitus and need for diagnostic adequacy. As such, our cumulative exposure metric represents a clinically realistic estimate of ionizing burden in this population.

Neurodevelopmental outcomes were evaluated using the Bayley Scales of Infant and Toddler Development, Third Edition (Bayley-III). Composite scores in the cognitive, motor, and language domains were assessed at 12–18 months corrected age during scheduled follow-up appointments at the Tufts Medical Center Neurodevelopmental Clinic. Trained developmental specialists performed all assessments as part of routine standardized follow-up care. At our institution, Bayley III assessments are conducted by certified developmental specialists trained in standardized administration of the tool. Tufts Medical Center is a reporting center for the Vermont Oxford Network (VON) Small Baby Database, and neurodevelopmental outcome data are collected and maintained under VON guidelines for quality and consistency.

The primary outcomes were the Bayley-III composite scores in the motor, cognitive, and language domains. To evaluate the relationship between cumulative x-ray exposure and neurodevelopmental performance, multiple linear regression analyses were conducted. Each model was adjusted for gestational age, birth weight, and SNAPPE-II score. Model diagnostics were performed to verify assumptions of linearity, normality of residuals, and absence of multicollinearity, including the calculation of variance inflation factors (VIF). Statistical analyses were performed using SPSS software, and a *p*-value of less than 0.05 was considered statistically significant.

## 3 Results

In our study, 53 preterm infants met the inclusion criteria. Table 1 summarizes the demographic and clinical characteristics of the study population. The mean gestational age was 29.2 ± 2.7 weeks, and the mean birth weight was 1,206 ± 419 grams. Among the cohort, 53% were delivered via Cesarean section, 43% were female, and 38% were identified as small for gestational age (SGA). Maternal characteristics showed that 46% of mothers had a recorded diagnosis of preeclampsia, 88% received at least one dose of antenatal betamethasone, and 48% received antenatal magnesium sulfate. At 12–18 months of corrected age, neurodevelopmental assessments using the Bayley Scales of Infant and Toddler Development, Third Edition (Bayley-III), yielded mean composite scores of 90.5 ± 10.9 in the motor domain, 89.7 ± 11.8 in the cognitive domain, and 83.4 ± 16.0 in the language domain.

**Table 1:** Demographic, clinical, and neurodevelopmental characteristics of the study cohort (*N* = 53).

Clinical parameter	Value
Gestational age (weeks)	29.2 ± 2.7
Birth weight (grams)	1,206 ± 419
Cesarean delivery (%)	53%
Female (%)	43%
Small for gestational age (SGA) (%)	38%
Maternal Diabetes (All causes)	46%
Maternal Chorioamnionitis	28%
Maternal preeclampsia (%)	46%
Antenatal betamethasone (%)	88%
Antenatal magnesium sulfate (%)	48%
1-min Apgar score [median (IQR)]	6 [4–7]
5-min Apgar score [median (IQR)]	8 [7–9]
Multiple gestation (%)	22%
Bayley-III Motor Score (mean ± SD)	90.5 ± 10.9
Bayley-III Cognitive Score (mean ± SD)	89.7 ± 11.8
Bayley-III Language Score (mean ± SD)	83.4 ± 16.0

SGA, small for gestational age (birth weight <10th percentile for gestational age).

Values are presented as mean ± SD or *n* (%) unless otherwise noted.

Apgar scores are presented as median [interquartile range].

**Figure 1:**
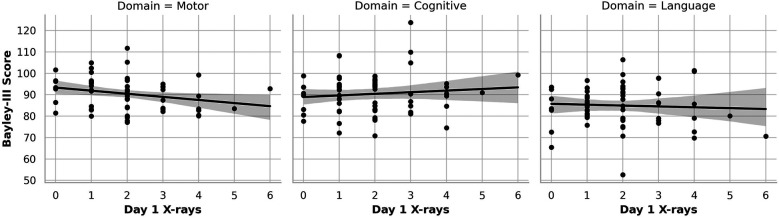
Association of cumulative Day 1 x-ray exposure with neurodevelopmental outcomes. This figure presents scatter plots with linear regression lines showing the relationship between cumulative diagnostic x-ray exposure on Day 1 of life and BSID-III composite scores at 12–18 months corrected age. Each panel represents a separate neurodevelopmental domain: motor (left), cognitive (center), and language (right). No statistically significant associations were observed for Day 1 x-ray exposure across any domain.

**Figure 2:**
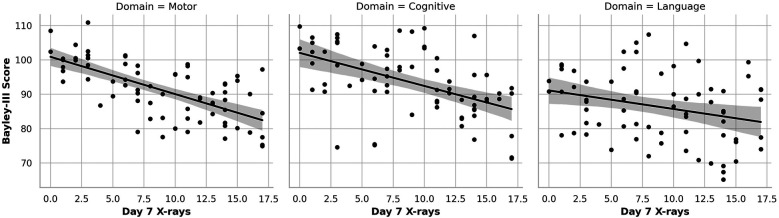
Association of Cumulative Day 7 x-ray Exposure with Neurodevelopmental Outcomes. Scatter plots with linear regression lines depict the association between cumulative diagnostic x-ray exposure by Day 7 of life and BSID-III composite scores at 12–18 months corrected age across three domains: motor (left), cognitive (center), and language (right). A significant inverse association is observed for motor and cognitive scores, with each additional x-ray linked to lower performance. Language scores show a downward trend, though not statistically significant.

**Figure 3:**
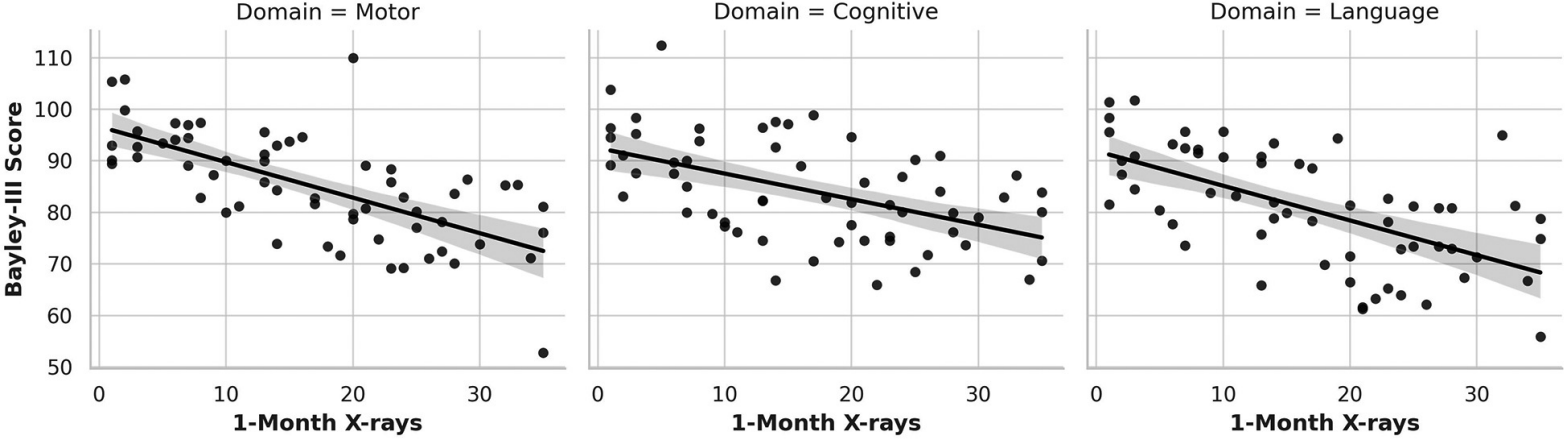
Association of Cumulative Day 30 x-ray Exposure with Neurodevelopmental Outcomes. This figure illustrates the relationship between cumulative x-ray exposure by one month of life and BSID-III neurodevelopmental outcomes at 12–18 months corrected age. Separate scatter plots show significant inverse associations for motor (left) and cognitive (center) domains, with higher imaging exposure linked to lower scores. Language outcomes (right) display a downward trend that did not reach statistical significance.

Figures 1–3 show the relationship between cumulative x-ray counts and all three domains of BSID motor, Cognitive and Language scores across different study time points, on Day 1(Figure 1), Day 7 (Figure 2) and Day 30 (Figure 3) respectively. x-ray imaging exposure was most limited on Day 1 (Figure 1) but increased substantially by Day 7 and Day 30 of life (Figuress 2,3). No significant association was found between x-ray exposure on Day 1 and neurodevelopmental outcomes in any domain (Figure 1). Multiple linear regression analyses adjusting for gestational age, birth weight, and illness severity (measured by SNAPPE-II) confirmed that x-rays performed within the first 24 h of life were not predictive of motor (*β* = –0.13, *p* = 0.899), cognitive (*β* = –0.11, *p* = 0.934), or language (*β* = 0.02, *p* = 0.918) scores.

In contrast, cumulative x-ray exposure by Day 7 (Figure 2) was significantly associated with lower motor and cognitive outcomes (Figure 2 left and middle graph). Each additional x-ray performed by Day 7 correlated with a 1.38-point decrease in Bayley-III motor scores (*p* < 0.001) and a 0.89-point decrease in cognitive scores (*p* = 0.046). Language scores (Figure 2 right graph) also trended downward (*β* = –0.43), although the association did not reach statistical significance (*p* = 0.129). This pattern continued at Month 1 (Figure 3), where cumulative imaging was again significantly associated with lower developmental scores. Motor scores (Figure 3, left graph) declined by 0.93 points per additional x-ray (*p* < 0.001), and cognitive scores (Figure 3, middle graph) declined by 0.78 points (*p* = 0.031). Language scores (Figure 3, right graph) continued to show a negative trend (*β* = –0.31, *p* = 0.172), though not statistically significant.

**Figure 4:**
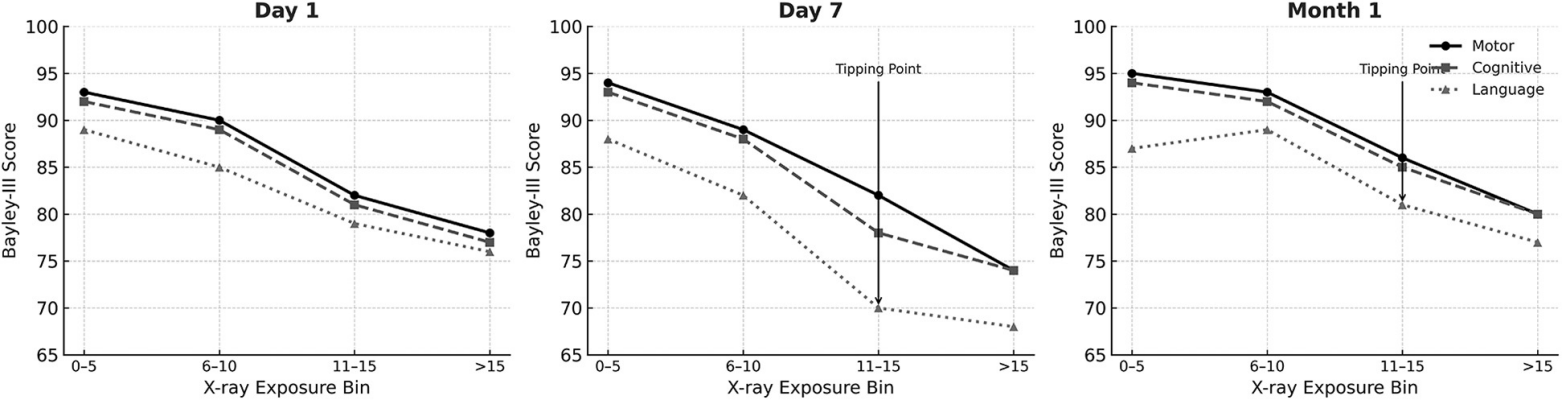
Bayley score trends by cumulative x-ray exposure at Day 1, Day 7, and month 1. Line graphs show the average BSID-III composite scores (motor, cognitive, and language) plotted against cumulative chest and abdominal x-ray counts at three time points: Day 1 (left), Day 7 (center), and 1 Month (right). A negative trend in motor and cognitive scores is evident with increasing imaging exposure, particularly beyond Day 7 and into the first month of life. Language scores also show a decline, though the association is less consistent.

Trend Analysis (Figure 4) conducted across Day 1, Day 7 and Month 1 shows the progression of developmental score decline with increasing imaging exposure over time further supporting the regression findings. At Day 1 (Figure 4, left graph), Bayley-III motor, cognitive, and language scores remained stable across different levels of imaging exposure, suggesting limited impact from early, minimal imaging. By Day 7 (Figure 4, middle graph), however, a clear decline in motor and cognitive scores was observed with increasing cumulative imaging, particularly among infants who had received more than 10 x-rays. This suggests a possible threshold effect beyond which neurodevelopmental risk becomes more pronounced. At one Month, this association strengthened, with the steepest declines observed in infants who had received more than 15 x-rays. (Figure 4, right graph). These infants exhibited the lowest average scores in both motor and cognitive domains. While language scores also declined with increasing exposure, the associations were more variable and less robust.

## 4 Discussion

Medical imaging plays a critical role in NICU care, helping to diagnose and monitor conditions such as respiratory distress syndrome (RDS), sepsis, necrotizing enterocolitis (NEC), and hemodynamic instability. However, concerns regarding the safety of repeated x-ray exposure in neonates have been growing.

This study provides new evidence that cumulative exposure to diagnostic x-rays in the first month of life is significantly associated with lower neurodevelopmental scores in preterm infants, specifically within the motor and cognitive domains of the BSID III scale. These associations persisted even after adjusting for gestational age, birth weight, and illness severity as measured by the SNAPPE-II score. Language outcomes also showed a negative trend with increasing imaging exposure, although these associations were weaker and not statistically significant. The trend analysis further reinforced a potential dose-dependent relationship, with sharper declines in developmental scores observed in infants who underwent more than 10–15 x-rays during their over the first month of life. Further, the average gestational age in our cohort was 29 weeks. It is likely that our findings are even more relevant for infants born at earlier gestational ages at an even more vulnerable time in neurodevelopment.

### 4.1 Long-term outcomes of early life radiation exposure

There is limited research that has evaluated radiation exposure in NICU settings. Weiß et al. (2) conducted a study quantifying radiation exposure from thoracic and abdominal radiographs in neonates, estimating potential cancer risks associated with cumulative imaging. They found that cumulative doses exceeded internationally recommended safety levels in certain high-risk populations (17, 18, 20, 22, 23). Similarly, Toscan et al. (7) highlighted that neonatal radiation exposure is often underestimated, with actual exposure levels likely higher than recorded. Crealey et al. (1) performed an audit of NICU imaging and found that very low birth weight (VLBW) and extremely low birth weight (ELBW) infants received significantly higher exposure to x-rays. They reported that these infants often undergo multiple radiographs per day, a trend also seen in our cohort, particularly among those with severe medical complications. Gilley et al. further emphasized the need for weight-based diagnostic reference levels (DRLs) to reduce unnecessary radiation exposure while maintaining diagnostic accuracy (8). Our study aligns with these concerns, showing that cumulative imaging at Day 7 and Month 1 was strongly associated with neurodevelopmental delays.

### 4.2 Neurodevelopmental impact of radiation exposure

Pearce et al. conducted a retrospective cohort study on pediatric CT scans and found that cumulative exposure increased the risk of leukemia and brain tumors (6). While our study does not explore oncogenic risks, the potential neurological consequences of repeated low-dose radiation remain a critical concern (19, 21, 25, 28). Our study provides new evidence linking cumulative and dose-dependent neonatal x-ray imaging with developmental delays, particularly in motor and cognitive domains, where higher imaging exposure correlates with greater neurodevelopmental impairment. Brody et al. emphasized that pediatric health providers should adopt an “Image Gently” approach to reduce unnecessary imaging (9, 10). This aligns with our recommendation to optimize imaging protocols to limit exposure in neonates at risk for developmental delays.

### 4.3 Potential mechanisms of neurodevelopmental impact

#### 4.3.1 Direct radiation-induced neurotoxicity

Radiation exposure is known to alter cellular function, particularly in rapidly dividing tissues such as the developing brain. Studies in pediatric radiation biology have shown that even low-dose radiation can disrupt neuronal growth, synaptogenesis, and white matter integrity (10, 11). Further, Brenner et al. found that low dose ionizing radiation exposure increased the risk of developmental disorders in pediatric populations. The developing brain is highly susceptible to external influences, including ionizing radiation, which can lead to oxidative stress and DNA damage. Studies suggest that the hippocampus and prefrontal cortex, regions essential for memory and cognitive function, may be particularly vulnerable to low-dose radiation (19, 25, 28). This is consistent with our findings of cognitive delays in relation to cumulative x-ray imaging.

Both chest and abdominal x-rays can contribute to scattered ionizing radiation, which may reach distant tissues such as the brain. Although abdominal x-rays are associated with lower cranial scatter compared to chest imaging, their cumulative effects—especially when frequent or overlapping—may still impact developing neural structures. Trinh et al. demonstrated measurable scatter to the cranial vault even from lower thoracic or abdominal imaging, raising concerns about inadvertent cerebral exposure in neonates despite attempts at field restriction (12). Our study did not distinguish between the two fields in terms of neurodevelopmental impact, but this represents an important area for future investigation.

#### 4.3.2 Dose–response and threshold patterns

One of the most striking observations in this study is the emergence of a dose–response pattern, particularly visible in the trend analyses starting at Day 7. This effect was most evident in infants who received more than 10 x-rays by this point—a pattern that continued and deepened by Month 1, where infants receiving over 15 x-rays exhibited the lowest average developmental scores. While the Bayley III scale provides early insights into neurodevelopment, scores obtained during infancy—particularly before 12 months—are known to have modest predictive validity for long-term cognitive and language outcomes. As such, our findings should be interpreted as reflective of early developmental trends rather than definitive prognostic indicators.

These findings are consistent with radiation safety literature, which suggests cumulative biological effect thresholds even for low-dose exposures, particularly when repeated frequently and in rapid succession (19–25, 27). The concept of a threshold or tipping point is clinically significant, as it implies that while some imaging may be necessary and safe, surpassing a certain frequency may sharply increase the risk of neurodevelopmental impairment. Our data do not pinpoint a precise threshold but suggest that clinical caution is warranted once imaging exceeds 10–15 exposures in the first month of life.

Trend analyses also revealed that the slope of decline steepened between Day 7 and Month 1, supporting the hypothesis of progressive injury with ongoing exposure. The fact that this pattern remained robust across adjusted regression models strengthens the argument that imaging frequency may have a cumulative and possibly irreversible effect on developing brain structures (13).

### 4.4 Indirect effects: medical fragility and increased interventions

While our study adjusted for major neonatal morbidities (IVH, HsPDA, sepsis, High grade IVH, Necrotizing enterocolitis), it is possible that frequent imaging reflects greater medical fragility rather than direct radiation effects. Infants requiring frequent imaging are often critically ill, requiring prolonged NICU stays, invasive procedures, and mechanical ventilation, all of which may contribute to neurodevelopmental delays (14–16). Increased medical interventions often lead to higher stress levels and sensory deprivation, which can impair cognitive and motor development.

While our models adjusted for SNAPPE-II as a measure of early illness severity, we could not capture NICU stress exposure or the cumulative burden of interventions, which may mediate part of the observed effects (26, 27). Nonetheless, the strength and persistence of associations across adjusted models suggest that imaging exposure itself is not merely a proxy for sickness, but a plausible contributor to developmental risk.

### 4.5 Limitations and future research directions

While this study offers valuable insights into the potential relationship between cumulative x-ray exposure and neurodevelopmental outcomes in preterm infants, several limitations should be acknowledged. First, the retrospective design limits the ability to establish causality. Although adjusted regression models revealed strong associations between imaging frequency and developmental outcomes, prospective cohort studies are needed to better understand the temporal and causal nature of this relationship. While the findings out to 1 month of age and cumulative x-ray exposure are striking, we did not evaluate degree of x-ray exposure after one month of age. However, as the average gestational age of infants in our study was 29 weeks, making their corrected gestational age approximately 34 weeks at one month of life. By this time-point, most infants are typically past the acute phase requiring frequent radiographic evaluation. However, we acknowledge this as a potential source of bias, particularly for infants with prolonged hospitalizations, and future studies should assess cumulative imaging exposure over the full NICU stay.

Second, the sample size of 53 infants, while adequate for exploratory analysis, limits the generalizability of the findings. This sample size was constrained by our rigorous inclusion criteria, including the exclusion of infants with incomplete follow-up (loss to follow up was 46%). However, given that families with less developmental concerns for their infants are more likely to not adhere to reminders for developmental testing, our findings may have been even more striking if our loss to follow up was less. Larger, multicenter studies are necessary to validate these results and to examine whether the observed associations persist across more diverse patient populations and care settings.

Third, this study did not include direct measurements of radiation dose. Instead, cumulative imaging counts were used as a surrogate for exposure, which limits the precision of dose-response analysis. Variations in radiographic technique, field size, shielding, and positioning were not accounted for and may contribute to variability in actual exposure between patients. An additional limitation of our study is the inability to distinguish between chest and abdominal radiographs, as imaging events were recorded cumulatively without anatomic stratification. In the neonatal population—especially among preterm infants—field overlap is common due to limited thoracoabdominal surface area and the need to ensure diagnostic coverage. Consequently, even when imaging is intended for a specific organ system, incidental exposure of adjacent structures, including the brain, can occur. This anatomical overlap limits the precision with which we can assign differential neurodevelopmental risk to specific imaging types. Future studies employing direct dosimetry or anatomical field classification could better delineate organ-specific radiation risks.

Fourth, several potentially important confounding variables were not included in the analysis. These include factors such as NICU environmental stress, the duration and invasiveness of interventions, parental involvement, and socioeconomic status, each of these may influence early neurodevelopmental trajectories and could interact with or confound the effects of cumulative imaging exposure. Another limitation relates to the use of SNAPPE-II scores as a measure of illness severity. While SNAPPE-II is widely validated and useful for early risk stratification, it is based solely on data from the first 12 h of life and may not fully capture evolving clinical status, prolonged instability, or cumulative stressors experienced by infants over time. Additionally, it does not incorporate key factors such as duration of mechanical ventilation, exposure to sedatives, or recurrent infections, which may also impact neurodevelopment. The use of Bayley III assessments between 6 and 12 months of corrected age presents inherent limitations, as developmental trajectories in preterm infants can change significantly with age. Longer-term follow-up beyond 24 months is necessary to confirm the persistence and clinical significance of the early differences observed.

Future research should aim to address these limitations. Longitudinal studies following preterm infants into early childhood and school age would help to determine whether early imaging-related neurodevelopmental differences persist or evolve over time. Larger studies with direct dosimetry are also needed to quantify radiation exposure more accurately and to refine our understanding of threshold effects. Additionally, research exploring the implementation of alternative imaging strategies, such as point-of-care ultrasound (POCUS) or low-dose protocols, would be valuable in assessing whether reductions in imaging frequency can improve long-term neurodevelopmental outcomes without compromising diagnostic accuracy.

### 4.6 Clinical and policy implications

Despite these limitations, the findings from this study hold substantial clinical relevance. The evidence for a dose-dependent association between imaging frequency and adverse neurodevelopment supports a shift toward more judicious use of diagnostic radiography in preterm infants. Clinicians should adopt the principle of ALARA (As Low as Reasonably Achievable) in decision-making, reserving imaging for instances where diagnostic yield justifies potential risk.

Moreover, this study adds weight to ongoing calls for the implementation of weight-based Diagnostic Reference Levels (DRLs) in neonatal imaging, as recommended by Gilley et al. (8). Institutional protocols should be updated to include automated tracking of imaging frequency, alerts for cumulative exposure thresholds, and preferential use of non-ionizing modalities, such as point-of-care ultrasound, wherever clinically appropriate (24, 27).

While the use of diagnostic x-rays in neonatology has declined in some centers due to increasing availability of safer modalities such as point-of-care ultrasound (POCUS) and functional near-infrared imaging (fNIRI), conventional radiography remains a mainstay in many NICUs, especially in settings with limited access to real-time ultrasound or operator expertise. Furthermore, line verification, respiratory assessments, and abdominal evaluations continue to rely on radiographs in the acute setting. Our findings reflect this current clinical reality and underscore the importance of accelerating the transition toward non-ionizing imaging protocols. By empirically demonstrating the dose–response relationship between cumulative radiography and neurodevelopmental outcomes, this study provides quantitative justification for expanding the implementation of POCUS and other safer diagnostic strategies in neonatal care. The integration of point-of-care ultrasound (POCUS) into routine NICU workflows offers a promising and practical alternative to diagnostic radiography. POCUS allows for bedside assessment of key organ systems—particularly the lungs, heart, and abdomen—without exposing vulnerable neonates to ionizing radiation. It has been increasingly validated for common neonatal indications such as line placement, pulmonary conditions, and intracranial assessment. Expansion of POCUS training programs and credentialing standards for neonatologists could accelerate adoption. Embedding POCUS within clinical decision pathways can not only reduce the need for radiography but also empower bedside providers with real-time, actionable data.

From a systems standpoint, radiation stewardship programs in NICUs should mirror successful antimicrobial stewardship models, with oversight committees, audits, and feedback mechanisms. Importantly, infants who have undergone frequent imaging (>10–15 x-rays in the first month) should be flagged for enhanced neurodevelopmental surveillance post-discharge. Early identification of delays, coupled with access to occupational, speech, and physical therapy services, may help mitigate long-term functional impairments.

## 5 Conclusion

This study demonstrates that cumulative x-ray exposure during the first month of life is associated with measurable declines in motor and cognitive development in preterm infants, particularly with repeated imaging beyond the first week of life. While early radiography remains necessary in certain clinical situations, our findings highlight the neurodevelopmental risks of frequent ionizing exposure.Although non-ionizing modalities like point-of-care ultrasound (POCUS) are increasingly available, conventional radiography continues to be widely used, especially in acute care and settings where ultrasound expertise is still evolving. Our results reinforce the urgent need to integrate POCUS more broadly into routine neonatal care—not only as a diagnostic tool, but as a strategy to minimize radiation exposure during a critical window of brain development.

Wider implementation of POCUS, supported by targeted training and institutional protocols, may substantially reduce reliance on radiography without compromising diagnostic accuracy. Future research should build on our findings by using direct dosimetry, assessing long-term outcomes, and evaluating the impact of POCUS-based imaging protocols on neurodevelopment. Until then, efforts to reduce imaging frequency, adopt ALARA principles, and prioritize POCUS integration should be core components of neonatal imaging stewardship.

## Data Availability

The data analyzed in this study is subject to the following licenses/restrictions: The dataset used in this study contains identifiable patient-level information collected from electronic medical records and is not publicly available due to institutional and ethical restrictions. Access may be granted upon reasonable request and with appropriate IRB and data-sharing approvals from Tufts Medical Center. Requests to access these datasets should be directed to indrani.bhattacharjee@tuftsmedicine.org.

## References

[1] Crealey M McCallion N Kempley S Bowden L Ludusan E Pathan M Utilization of conventional radiography in a regional neonatal intensive care unit in Ireland. J Matern Fetal Neonatal Med. (2019) 32(16):2667–73. 10.1080/14767058.2018.1445713 29478349

[2] Weiß D Beeres M Rochwalsky U Vogl TJ Schlößer R. Radiation exposure and estimated risk of radiation-induced cancer from thoracic and abdominal radiographs in 1307 neonates. Eur Radiol. (2025) 35(1):297–308. 10.1007/s00330-024-10942-x 39014087 PMC11632034

[3] Bushberg JT Seibert JA Leidholdt EM Jr Boone JM. The Essential Physics of Medical Imaging. 3rd ed. Philadelphia: Lippincott Williams & Wilkins (2011).

[4] Antonelli F Casciati A Belles M Serra N Linares-Vidal MV Marino C Long-term effects of ionizing radiation on the hippocampus: linking effects of the sonic hedgehog pathway activation with radiation response. Int J Mol Sci. (2021) 22(22):12605. 10.3390/ijms222212605 34830484 PMC8624704

[5] Kleinerman RA. Cancer risks following diagnostic and therapeutic radiation exposure in children. Pediatr Radiol. (2006) 36(2):121–5. 10.1007/s00247-006-0191-5 16862418 PMC2663653

[6] Pearce MS Salotti JA Little MP McHugh K Lee C Kim KP Radiation exposure from CT scans in childhood and subsequent risk of leukaemia and brain tumours: a retrospective cohort study. Lancet. (2012) 380(9840):499–505. 10.1016/S0140-6736(12)60815-0 22681860 PMC3418594

[7] Toscan M de Araújo BF Martini JC Ravazio R de Souza VC. Our estimates of neonatal radiation exposure fall short of reality. Eur J Pediatr. (2024) 183(4):1911–6. 10.1007/s00431-024-05466-x 38334796

[8] Gilley R David LR Leamy B Moloney D Moore N England A Establishing weight-based diagnostic reference levels for neonatal chest x-rays. Radiography. (2023) 29(4):812–7. 10.1016/j.radi.2023.05.012 37276688

[9] Smans K Struelens L Bosmans H Vanhavere F. Dosimetry of neonatal chest radiography using MOSFET detectors. Radiat Prot Dosimetry. (2006) 120(1–4):17–20.

[10] Satharasinghe DM Jeyasugiththan J Wanninayake WM Pallewatte AS. Paediatric diagnostic reference levels in computed tomography: a systematic review. J Radiol Prot. (2021) 41(1):R1. 10.1088/1361-6498/abd840 33684071

[11] Brody AS Frush DP Huda W Brent RL, Section on Radiology. Radiation risk to children from computed tomography. Pediatrics. (2007) 120(3):677–82. 10.1542/peds.2007-1910 17766543

[12] Trinh AM Schoenfeld AH Levin TL. Scatter radiation from chest radiographs: is there a risk to infants in a typical NICU? Pediatr Radiol. (2010) 40(5):704–7. 10.1007/s00247-009-1474-4 19997727

[13] Balentova S Adamkov M. Molecular, cellular and functional effects of radiation-induced brain injury: a review. Int J Mol Sci. (2015) 16(11):27796–815. 10.3390/ijms161126068 26610477 PMC4661926

[14] Perveen S Kakkilaya V Alonso-Ojembarrena A Shin SH Hwang JK Shin SH Bronchopulmonary dysplasia: latest advances. Front Pediatr. (2023) 11:32. 10.3389/fped.2023.1303761 PMC10679731 38027276

[15] Frush DP Donnelly LF Rosen NS. Computed tomography and radiation risks: what pediatric health care providers should know. Pediatrics. (2003) 112(4):951–7. 10.1542/peds.112.4.951 14523191

[16] McParland BJ. A study of patient radiation doses in neonatal intensive care unit radiography. Br J Radiol. (1998) 71(847):728–33. 10.1259/bjr.71.842.95791829771383

[17] Toossi MT Malekzadeh M. Radiation dose to newborns in neonatal intensive care units. Iran J Radiol. (2012) 9(3):145. 10.5812/iranjradiol.8065 23329980 PMC3522370

[18] Osei EK Darko J. Foetal radiation dose and risk from diagnostic radiology procedures: a multinational study. Int Sch Res Notices. (2013) 2013(1):318425. 10.5402/2013/318425 PMC4045527 24959554

[19] Kovalchuk A Kolb B. Low dose radiation effects on the brain–from mechanisms and behavioral outcomes to mitigation strategies. Cell Cycle. (2017) 16(13):1266–70. 10.1080/15384101.2017.1320003 28656797 PMC5531620

[20] Dougeni ED Delis HB Karatza AA Kalogeropoulou CP Skiadopoulos SG Mantagos SP Dose and image quality optimization in neonatal radiography. Br J Radiol. (2007) 80(958):807–15. 10.1259/bjr/77948690 17875594

[21] Nelson TR. Practical strategies to reduce pediatric CT radiation dose. J Am Coll Radiol. (2014) 11(3):292–9. 10.1016/j.jacr.2013.10.011 24589405

[22] Ryu JH Shin SH Choi YH Kim EK Kim HS. Minimizing radiation exposure in neonatal intensive care unit: a quality improvement approach on x-ray practices. Neonatal Med. (2024) 31(3):56–64. 10.5385/nm.2024.31.3.56

[23] Miglioretti DL Johnson E Williams A Greenlee RT Weinmann S Solberg LI The use of computed tomography in pediatrics and associated radiation exposure. JAMA Pediatr. (2013) 167(8):700–7. 10.1001/jamapediatrics.2013.311 23754213 PMC3936795

[24] Goske MJ Charkot E Herrmann T John SD Mills TT Morrison G Image gently: challenges for radiologic technologists when performing digital radiography in children. Pediatr Radiol. (2011) 41:611–9. 10.1007/s00247-010-1957-3 21491201

[25] Brenner DJ Hall EJ. Computed tomography: an increasing source of radiation exposure. N Engl J Med. (2007) 357(22):2277–84. 10.1056/NEJMra072149 18046031

[26] Goldwein JW. Effects of radiation therapy on skeletal growth in childhood. Clin Orthop Relat Res. (1991) 262:101–7. 10.1097/00003086-199101000-00014 1984904

[27] Stewart DL Elsayed Y Fraga MV Coley BD Annam A Milla SS. Use of point-of-care ultrasonography in the NICU for diagnostic and procedural purposes. Pediatrics. (2022) 150(6):e2022060053. 10.1542/peds.2022-060053 37154781

[28] Monje ML Mizumatsu S Fike JR Palmer TD. Irradiation induces neural precursor-cell dysfunction. Nat Med. (2002) 8(9):955–62. 10.1038/nm749 12161748

